# Bacterial Outer Membrane Vesicles: Role in Pathogenesis and Host-Cell Interactions

**DOI:** 10.3390/antibiotics13010032

**Published:** 2023-12-28

**Authors:** Gisseth Magaña, Caitlyn Harvey, Clifford C. Taggart, Aoife M. Rodgers

**Affiliations:** Wellcome Wolfson Institute for Experimental Medicine, School of Medicine, Dentistry and Biomedical Sciences, Queens University Belfast, Belfast BT9 7AE, UK; gmaganazapata01@qub.ac.uk (G.M.); charvey10@qub.ac.uk (C.H.); c.taggart@qub.ac.uk (C.C.T.)

**Keywords:** outer membrane vesicles, pathogenesis, OMV biogenesis, Gram-negative bacteria

## Abstract

Outer membrane vesicles (OMVs) are small, spherical structures released from the outer membranes of Gram-negative bacteria into the surrounding environment. Investigations into OMVs range from their biogenesis and cargo composition to their ability to transfer virulence factors and modulate host immune responses. This emerging understanding of OMVs has unveiled their pivotal role in the pathogenicity of infectious diseases, shedding light on their interactions with host cells, their contributions to inflammation, their potential involvement in antimicrobial resistance, and their promising use for the development of novel treatments and therapies. Numerous studies have associated the OMVs of pathogenic bacteria with the exacerbation of inflammatory diseases, underlining the significance of understanding the mechanisms associated with these vesicles to find alternatives for combating these conditions. Additionally, OMVs possess the ability to act as decoys, absorbing and neutralizing antibiotics, which significantly diminishes the efficacy of a broad spectrum of antimicrobial agents. Another subtopic of interest is OMVs produced by commensal microbiota. These vesicles are increasingly acknowledged for their mutualistic functions, significantly influencing their host’s physiology and immune responses. Consequently, OMVs play a crucial role in maintaining a balanced gut microbiota by fostering symbiotic relationships that significantly contribute to the overall health and well-being of the host. This comprehensive review aims to provide an up-to-date review of OMVs derived from Gram-negative bacteria, summarizing current research findings, and elucidating the multifaceted role of these vesicles in diverse biological contexts.

## 1. Introduction

The survival and adaptation of microorganisms within a host requires communication with the surrounding environment. The secretion of cellular compounds and extracellular vesicles (EVs) towards the exterior plays a crucial role in inter- and intra-species interactions in all domains [1,2,3]. Bacteria have the ability to secrete a wide range of vesicles, commonly known as bacterial membrane vesicles (MV), that can vary in their content and function [4]. The secretion of vesicles has been studied in both Gram-negative and Gram-positive bacterial species [5]. While these vesicles are referred to as outer membrane vesicles (OMVs) in Gram-negative bacteria, in Gram-positive bacteria, MVs are known as cytoplasmic membrane vesicles (CMV) [5,6].

The variation in MV composition arises from differences in the structures of bacterial membranes. However, Gram-positive MVs carry a wide array of molecules that resemble those found in Gram-negative microorganism MVs, although with significant distinctions [2,5]. The biogenesis of MVs in Gram-positive microorganisms originates from the cytoplasmic membrane, in contrast to their Gram-negative analog, which primarily originates from the outer membrane (OM) [6,7]. These differences in membrane origin result in variations in the composition of their respective vesicles. For instance, Gram-positive MVs notably lack lipopolysaccharides (LPS), a molecule that is exclusive to Gram-negative bacteria [8]. 

OMVs are small, spherical, and bilayered structures with diameters ranging from 20 to 350 nm that are released during Gram-negative bacterial phase growth [9,10]. OMVs have been observed for all Gram-negative bacterial strains studied to date, including *Pseudomonas aeruginosa*, *Vibro cholerae*, *Neisseria Meningitidis*, *Neisseria gonorrhoeae*, *Salmonella enterica serovar Typhimurium*, *Bacteroides fragilis*, *Helicobacter pylori*, and *Haemophilus influenzae* [10,11]. Studies have demonstrated that OMVs comprise a wide range of biomolecules and a high concentration of toxins and virulence factors, enabling cell-to-cell communication with the host and initiating pathogenesis, even in the absence of live bacteria [12,13]. OMVs have also been associated with the delivery of metabolites and elemental molecules for other bacterial species, including growth factors and anti-inflammatory substances [3,14,15].

*Haemophilus influenzae* is a Gram-negative bacterium commonly associated with pathogenic activity while colonizing the respiratory tract [16]. It can be classified based on the absence (non-typeable) or presence (typeable) of a polysaccharide capsule [16]. Non-typeable *Haemophilus influenzae* (NTHi) is one of the most common bacteria associated with respiratory diseases such as pneumonia, sinusitis, and otitis media [17]. NTHi utilises several sophisticated mechanisms to colonize and infect airway epithelial cells and macrophages from the host, including the release of OMVs [18]. NTHi-OMVs can transport a wide range of cargo molecules, including proteins, LPS, and phospholipids, which are recognised by specific host cell receptors [19]. NTHi-OMVs can induce pro-inflammatory responses in the host, resulting in the release of cytokines and chemokines [18]. 

In recent years, the study and understanding of bacterial OMVs have increased and gained relevance in the health sector due to their potential use as long-distance transporter molecules that can be used as vaccines and novel therapeutic options. OMVs’ properties have been successfully tested in developing a vaccine to protect against *Neisseria gonorrohoeae* (*N. gonorrohoeae*) [20]. However, further research efforts are necessitated to unravel and elucidate the mechanisms of action of OMVs derived from different bacteria, in addition to the host immune response that they induce and their role in bacterial pathogenesis.

The present review provides an up-to-date account of the current knowledge of Gram-negative bacterial OMVs, with a particular focus on non-typeable *Haemophilus influenzae* (NTHi), and with respect to their biogenesis, role in bacterial pathogenesis, and host–cell interactions. 

## 2. OMV Cargo

Before understanding OMVs’ cargo composition, it is essential to understand the general structure of Gram-negative bacteria. The membrane structure of Gram-negative bacteria is composed of two membranes with diverse chemical and structural systems. First, the inner membrane (IM) comprises a fluid phospholipid bilayer and a peptidoglycan cell wall composed of repeating N-acetyl glucosamine-N-acetyl muramic acid units. The OM structure is composed of phospholipids in the inner leaflet and LPS in the outer leaflet which are anchored by nonspecific porins. The region compressed between the OM and IM is called the periplasm and consists of a watery compartment containing a wide variety of proteins [21]. The membrane structure is crucial to preserving high concentrations of cargoes and protecting them from degradation [22]. When the OMVs are expelled, they carry a highly diverse load of cargo molecules, including LPS, outer membrane proteins (OMPs), lipooligosaccharides (LOS), phospholipids, peptidoglycan, periplasmic elements, and virulence factors, including enzymes and toxins [12]. The presence of molecules such as deoxyribonucleic acid (DNA) and ribonucleic acid (RNA) has also been reported. However, nucleic acid packaging and its delivery into OMVs still needs to be unraveled [23,24,25]. 

The molecular cargo of OMVs can vary depending on the bacterial type, growth conditions, and environmental factors [12]. Proteins constitute one of the most abundant components in OMVs and play diverse functional roles. The emergence of technologies such as mass spectrometry (MS) over the last decade has enabled the identification of thousands of OMV-associated proteins [26]. One study in particular focused on Gram-negative bacteria’s constitutive secretion of native OMVs into the extracellular environment. In this proteomics-based study, researchers identified 141 protein components within *E. coli*-derived native OMVs [27].

OMVs have also been shown to contain OMPs, including porins such as the outer membrane proteins (Omp), OmpF, OmpA, and OmpC, in addition to anchoring proteins, which can be recognized by specific receptors on the host cells [23,28]. Such proteins can promote bacterial adhesion, thereby enhancing their ability to bind to host cells. The significant diversity observed in OMVs, which arise from a wide range of microorganisms, has led to the hypothesis that bacteria possess unique and precise sorting mechanisms for OMVs’ biogenesis, besides it being a byproduct of cell lysis. In contrast to a scenario in which OMVs could be perceived as outcomes of cellular decay, this hypothesis proposes that the varied composition of OMVs reflects an active and specific biological process. Bacteria appear to deliberately include different components in OMVs, underscoring the fundamental role of OMVs’ formation in bacterial physiology and environmental interactions [23,29].

## 3. OMV Biogenesis

OMVs’ biogenesis is a complex process influenced by various factors, including the external environmental and host conditions [30]. As an example, the enteric bacterial pathogen *Campylobacter jejuni* can sense host metabolites, such as sodium bile salts, which significantly influences both the production and content of OMVs. Bile is excreted from the gall bladder into the small intestine, whereby it functions to emulsify dietary fats and facilitates the absorption of lipid nutrients. A study by Elmi et al. (2017) delved into the influence of sodium bile salt on the production, content, and toxicity of OMVs produced by *Campylobacter jejuni*. Their study revealed that the presence of bile salt significantly influenced the genetic landscape associated with OMVs’ biogenesis. The OMVs produced in the presence of bile salt exhibited a distinct protein profile compared to those isolated in the absence of bile salt, signaling a direct link between the external environment and the genetic machinery governing vesicle formation. Consequently, it was determined that the production of OMVs in the presence of bile salt led to an elevation in the mRNA transcription levels of serine protease genes associated with OMVs’ biogenesis. Additionally, this external condition enhanced the cytotoxicity and immunogenicity of the produced OMVs toward intestinal epithelial cells [31].

**Figure 1:**
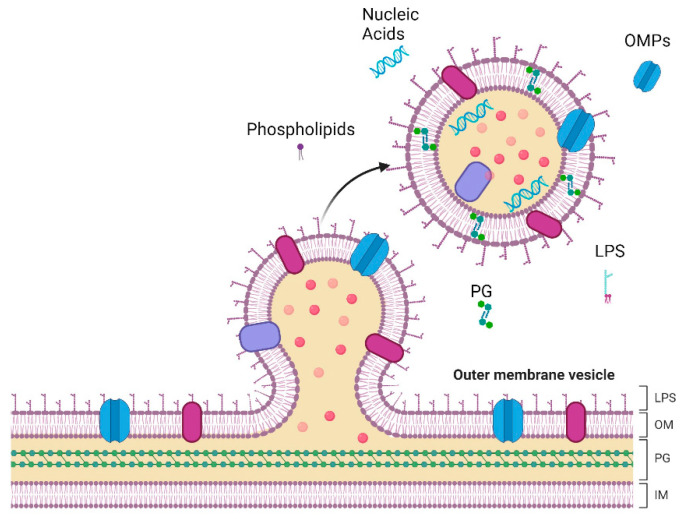
Representation of vesicle formation and release in the outer membrane (OM) of Gram-negative bacteria. The complex architecture of Gram-negative bacteria comprises the inner membrane (IM), a phospholipid bilayer, and a peptidoglycan (PG) cell wall. The OM is structured with phospholipids in the inner leaflet and lipopolysaccharides (LPS) in the outer leaflet, anchored by Outer Membrane Proteins (OMPs). These essential components are also present in Outer Membrane Vesicles (OMVs). Created with BioRender.com.

In a general overview, OMVs’ biogenesis begins with bulging of the OM and culminates in the release of vesicles into the surrounding milieu (Figure 1) [23]. However, there are still some gaps in understanding the molecular mechanism of OMVs’ formation, particularly regarding the genes involved [32]. Despite this, different models have attempted to elucidate the complex process of bacterial vesicle formation in various bacterial strains. The first observations of bacterial vesicles dates back to the 1960s. Even so, it was only in recent decades that a significant increase in reports on OMVs biogenesis, physiological functions, and applications occurred [33]. Knox et al. (1966) were among the pioneers, reporting the release of bacterial “vesicles” in the lysine-requiring mutant *Escherichia coli* (*E. coli*) 12,408. These “globules” were surrounded by membranes without showing evidence of cell lysis [34]. Subsequent studies involving different strains of *E. coli* showed that the extracted vesicles of live bacteria contained lipids, proteins, and LPS. Notably, these vesicles had compositions similar to those found in the OMs of whole cells [35,36]. These pioneering findings laid the foundation for subsequent research on the formation of OMVs [33].

Three models have been proposed to elucidate the role of lipoproteins, LPS, and peptidoglycan in the biogenesis of OMVs [33]. An overview of these models and new insights into the molecular determinants involved in OMV biogenesis are provided in this section. The first model suggests that OMVs form due to the limited number of lipoproteins that bind to the peptidoglycan layer. The scarcity of lipoprotein binding causes the OM to bulge, thus affecting the vesicles’ genesis. Hoekstra et al. (1976) conducted a study to determine the quantity of lipoproteins present in both the OM and OMVs of *E. coli.* Their study revealed that the vesicles contained fewer lipoproteins compared to the OM. Based on these findings, the authors proposed that OMVs are released from areas on the OM with limited lipoprotein binding [36,37]. 

Secondly, the following model is founded on the presence of peptidoglycan residues containing autolysins in OMVs. According to this model, specific sites exhibit higher peptidoglycan concentrations during the synthesis of the peptidoglycan layer, causing bulges in the OM. This initiates a chain of signals that leads to the formation of vesicles. Supporting evidence for this model came from observing muramic acid, a known precursor of the peptidoglycan layer, in OMVs purified from *Porphyromonas gingivalis* [38]. Furthermore, the mutation of an autolysin involved in peptidoglycan replacement increased OMV synthesis [39]. These findings indicate that the accumulation of peptidoglycan residues causes the bulging of the OM, leading to the release of vesicles [38,39].

The final model examines how the charge of LPS influences OMVs’ genesis. *Pseudomonas aeruginosa* is known to produce both negatively and neutrally charged LPS. The OMVs excreted by this bacterium, particularly under oxidative stress, are primarily composed of negatively charged LPS. Consequently, it has been proposed that the increased presence of negatively charged LPS within the cell envelope facilitates OMV release by the generation of repulsive forces due to the negative charges present in the OM [40,41]. These models underscore the significance of lipoproteins, LPS, and peptidoglycan during OMVs’ formation. However, it remains unknown whether these mechanisms act in concert.

In recent years, significant progress has been made in unraveling the genetic basis of OMV formation. A study by Nevermann et al. (2019) focused on understanding this process in *Salmonella enterica* serovar Typhi (*S.* Typhi). The researchers successfully identified nine genes, collectively called the “zzz genes,” which played crucial roles in the increase in HlyE toxin secretion and the biogenesis of OMVs. These zzz genes engender several functions, including envelope stability (*OmpA*, *nlpI*, *tolR*), lipopolysaccharide synthesis (*rfaE*, *waaC*), peptidoglycan synthesis and remodeling (*mrcB*), stress sensing (*degS*), and global transcriptional regulation (*hns*) [32]. This study represents a significant advancement in understanding OMVs’ biogenesis and sheds light on the specific genetic determinants involved in this process. The findings have potential implications for understanding bacterial pathogenesis, vaccine development, and advancing biotechnological applications [32].

## 4. Role of OMV Bacterial Pathogenicity

Due to the diverse array of biomolecules contained within OMVs, they play a pivotal role in several biological processes that enable bacteria to adapt to their environment, facilitating survival under stressful conditions, acquiring nutrients, and modulating host–pathogen interactions [23,42]. For example, OMVs can induce apoptosis in host cells by activating the immune response by administering cytotoxic factors, including cytokines that induce tissue damage [43,44]. Cecil et al. (2017) demonstrated that OMVs derived from *Porphyromonas gingivalis*, *Treponema denticola*, and *Tannerella forsythia* affect the activation of pro-inflammatory responses, including increasing the release of TNF-α and IL-8, along with the secretion of IL-1β and the activation of the ASC inflammasome. This leads to the activation of monocytes and macrophages, resulting in inflammatory cell death and tissue damage in vitro and in vivo following stimulation with OMVs derived from these bacteria [44].

OMVs have been strongly linked with pathogenesis due to their ability to enter epithelial cells and macrophages, which triggers an immediate innate immune response from the host that promotes inflammation [10]. This is facilitated by a long-distance delivery mechanism that enables bacterial invasion of the immune system, thereby promoting successful host colonization [23]. Bomberger et al. (2009) demonstrated that OMVs from *Pseudomonas aeruginosa* are not only involved in inflammation but also directly deliver multiple virulence factors to host airway epithelial cells, causing cytotoxic effects. Four key virulence factors, including alkaline phosphatase, β-lactamase, hemolytic phospholipase C, and Cif, were detected in the cytoplasm of airway epithelial cells after exposure to purified OMVs. Remarkably, these virulence factors were delivered into host cells in the absence of live bacteria, emphasizing the significance of OMVs in altering host cell physiology independently. This study revealed that OMVs play a crucial role in bacterial pathogenicity by serving as vehicles for the long-distance delivery of virulence factors to host cells [45].

**Figure 2:**
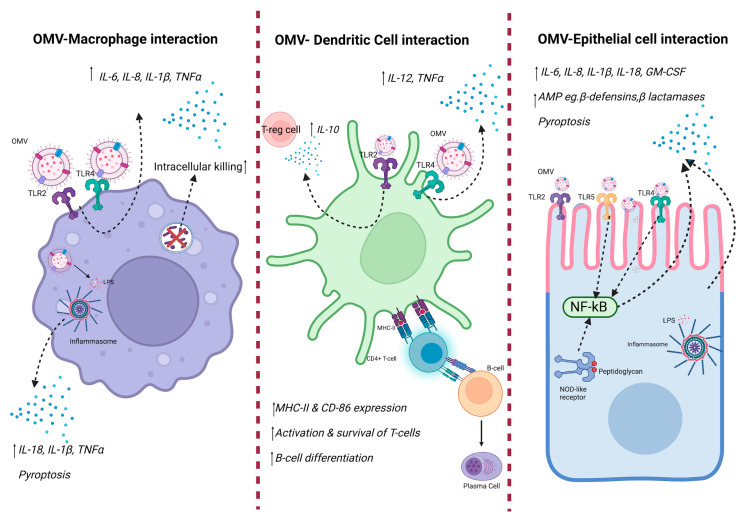
Innate immune response against OMV. OMVs contain DAMPs, including LPS, outer membrane proteins, and bacterial DNA from the bacteria they are derived from. Such DAMPs are recognized by host PRRs, resulting in downstream immune signaling, thus causing production of pro-inflammatory cytokines. Created with BioRender.com.

Live organisms have developed complex means to detect the invasion of external pathogens, including viruses, fungi, and bacteria. This process begins when immunological molecules, such as LPS, peptidoglycan, and flagellin, carried by OMVs, interact with the host’s receptors stimulating the immune response [23,46,47]. The initial detection of pathogens is led by germline-encoded pattern-recognition receptors (PRRs), which are responsible for recognizing pathogen-specific molecular signatures, also called pathogen-associated molecular patterns (PAMPs). Among the PPRs characterized to date are Toll-like receptors (TLRs), of which TLR1, 2, and 6 have been linked with the recognition of lipoproteins and peptidoglycans and TLR4 with LPS, while TLR3, 7, and 9 are related to binding nucleic acid sensors [48]. The binding of this complex is denominated as PRR-DAMPs/PAMPs and is involved in triggering immune responses and inflammation in the host [19]. Although this process is essential for pathogen elimination, uncontrolled inflammation can be detrimental, resulting in a double-edged sword by causing severe organ damage after the inflammation process [49]. Ultimately, OMVs couple the delivery of bacterial components and the generation of a highly pro-inflammatory environment in order to create a sustainable niche for bacteria to colonize and establish infection. As outlined in Figure 2, OMVs can activate multiple signaling pathways in macrophages, dendritic cells, and epithelial cells. 

## 5. Role of OMV in Inflammatory Diseases

In recent years, the contributions of OMVs to inflammatory diseases have been widely documented, particularly in conditions such as periodontal disease, gastrointestinal inflammation (including inflammatory bowel disease), and pulmonary inflammation [23,30,50]. The exact mechanism of how this occurs is yet to be defined; however, it is likely dependent on the bacteria from which the OMVs derive and also a cumulation of small changes, each contributing, rather than a definitive mechanism. The presence of ‘foreign’ particulate in the host leads to a general increase in inflammatory cytokines, which tends to heighten inflammation and cause exacerbations in pre-existing inflammatory conditions. Cargo carried within OMVs often contains virulent factors which not only increase pathogenicity by promoting bacterial adherence but also reduce the host’s ability to clear the pathogen. This results in increased susceptibility to colonization and the survival of bacteria, subsequently resulting in sustained inflammation. For example, in the context of periodontal disease, OMVs from *Fusobacterium nucleatum* have been observed to increase the number of osteoclasts and stimulate the release of inflammatory cytokines within the connective tissues of the gums. This exacerbates the symptoms associated with periodontitis [50], highlighting the pivotal role of OMVs in promoting periodontitis-associated inflammation. As a further example, in the intestine, OMVs originating from *Helicobacter pylori* which carry virulence factors are rapidly internalized by gastric epithelial cells, causing disruption of the protective mucin barrier and promoting bacterial colonization, leading to the progression of gastric diseases [51].

Several studies have reported that various strains of Gram-negative bacteria induce pro-inflammatory responses in different cell types, including epithelial cells, macrophages, and dendritic cells, upon the release of OMVs [30]. A recent study has shown that lung inflammation and macrophage pro-inflammatory responses are induced by bacterial-derived OMVs. Importantly, such responses are a result of multiple signaling pathways, not only that of TLR4/TLR2-mediated pathways induced upon interaction with LPS transported by OMVs [30]. Ryu et al. (2023) demonstrated that OMVs from *E. coli* robustly induce TLR1, TLR2, and TLR6 signaling, while significantly reducing TLR5 and TLR9. Unlike previous reports, TLR4 was not affected after interaction with OMVs in this study. To better understand this finding, cells lacking TLR4 were stimulated with OMVs. The outcomes showed a considerable reduction in the levels of LPS-induced pro-inflammatory cytokines upon TLR4 deletion. However, the OMV-treated cells were minimally affected. These results suggest that OMVs induce the activation of multiple signaling pathways and not only TLR4. Despite the novel findings of this study, further investigation of the activation and signaling pathways in other bacterial strains and the specific factors contained within OMVs inducing such responses warrants further investigation [30,52].

Further studies, conducted with the pathogen *Moraxella catarrhalis*, have provided insight into the pivotal role played by OMVs in activating tonsillar B cells from human tonsils [53]. While B cells are well-known for their crucial role in pathogen endocytosis and clearance, the study conducted by Perez et al. (2010) revealed, for the first time, that OMVs secreted by this bacterium actively facilitate the rescue and survival of pathogens. The interaction process is primarily initiated by the binding of the IgD receptor on B cells, followed by the release of calcium ions (Ca^2+^) and subsequent receptor internalization. In contrast to the findings of Ryu et al. (2023), it was observed that OMVs’ interaction with B cells primarily leads to the release of TLR9 and TLR2 [30,54]. 

NTHi is a Gram-negative bacterium belonging to the *coccobacillus* family that has been associated with causing a wide range of mucosal infections, including asthma, otitis media, sinusitis, meningitis, pneumonia, and exacerbations in patients with chronic obstructive pulmonary disease (COPD) [16,18,54]. NTHi has developed a series of complex and successful mechanisms that allow it to infect the upper and lower respiratory tract while evading the host immune system, including releasing OMVs [16,18]. One example of these mechanisms involves the abrogation of pathogen clearance by the host’s immune system [55]. This process involves acquiring complement regulatory proteins, such as the C4BP protein that binds to the C4 receptor and vitronectin that anchors factor H. This acquisition allows the pathogen to effectively suppress complement-mediated killing [55,56,57], promoting its survival within the host. 

Another extensively investigated pathogenetic factor in NTHi is the OMP 5 (P5), which belongs to the A family of proteins (OmpA). This protein exhibits a distinctive architecture, comprising two principal domains: the conserved N-terminal membrane-embedded β-barrel transmembrane domain, accompanied by four highly variable and immunogenic extracellular surface loops, denoted as loop 1–4, and a conserved periplasmic C-terminal domain (CTD) [58,59]. The multifunctional surface loops of P5 play a pivotal role in NTHi’s pathogenicity. Firstly, they contribute to the bacterium’s adherence to the host’s airway mucosa by binding to mucin, Intercellular Adhesion Molecule 1 (ICAM-1), and Carcinoembryonic Antigen-Related Cell Adhesion Molecule 1 (CEACAM1) receptors. This adhesion process facilitates colonization and infection. Secondly, these surface loops also play a crucial role in enhancing the pathogen’s resistance to the host’s immune defense mechanisms. Notably, P5 enables the acquisition of complement regulatory proteins, namely C4BP and FH (factor H), thereby providing the bacterium with a means to suppress complement-mediated killing [60,61,62]. 

Su et al. (2023) have also demonstrated that the C-terminal domain (CTD) of P5 may play a crucial role in OM stability and pathogenicity by promoting OMVs’ generation and conferring resistance to β-lactam antibiotics. Thus, a comparative analysis using various strains of NTHi was conducted. The researchers utilised the wild-type strain NTHi 3655 as a reference and generated variants, including NTHi 3655Δp5^CTD^ with a deletion in the C-terminal domain of the P5 protein and NTHi 3655Δp5::p5 with a truncated form of P5. A control strain, NTHi 3655Δp5, was also included, wherein the entire P5 protein was deleted. The results revealed that both NTHi 3655Δp5^CTD^ and 3655Δp5 (mutant strains with deletions in P5) produced significantly higher amounts of OMVs compared to that of the unmutated NTHi 3655 strain, suggesting a regulatory role of the C-terminal domain in OMVs’ formation. Furthermore, strains with C-terminal deletions were more susceptible to β-lactam antibiotics, including ampicillin and imipenem, compared to that of the NTHi 3655 strain. These findings indicate that the C-terminal domain in P5 is associated with increased bacterial resistance to these antibiotics [56], and, importantly, is associated with increased production in OMV.

The connection between the release of OMVs and NTHi pathogenicity has also been studied. Sharpe et al. (2011) identified 142 proteins present in OMVs derived from the 80-028NP strain of NTHi. Moreover, this group elucidated and proposed a binding model illustrating the interaction between OMVs and human pharyngeal cells that facilitates infection. This dynamic mechanism starts with the internalization of host cells with OMVs through caveola-dependent endocytosis that gives rise to the significant release of the immunomodulatory chemokine interleukin-8 (IL-8) and the antimicrobial peptide (AMP) LL-37 that is secreted from host epithelium as a response mechanism to bacterial colonization [62]. Additionally, the authors analyzed the role of OMVs’ surface proteins in stimulating IL-8 release. They achieved this by subjecting OMVs to proteinase K treatment to degrade surface-exposed proteins. The findings suggested that lipooligosaccharides play a pivotal role in modulating the inflammatory response of these vesicles. Moreover, the vesiculation process appears to function as a buffering mechanism against the host’s innate immune response, thereby protecting NTHi from pathogen elimination [62]. 

Zhang et al. (2023) investigated the pathogenesis of NTHi in promoting neutrophilic asthma through the inhalation of OMVs. Mice were nebulized with ovalbumin (OVA) and subsequently exposed to the inhalation of OMVs. Following this, lung tissues from mice treated with OVA+NTHi OMVs were analyzed by immunohistochemical approaches, revealing a significant disruption in the morphology of respiratory epithelial cells, with increased inflammation around the bronchioles. Additionally, analysis of pro-inflammatory cytokines revealed increased levels of IL-1β, tumor necrosis factor (TNF)-α, and IL-6 in bronchoalveolar lavage fluid (BALF), compared to that of the control group. This suggests that inhaling NTHi OMVs in OVA-sensitized mice induces airway inflammation [18]. Blood and BALF samples were also analyzed to determine whether OVA + NTHi-treated mice exhibited typical indicators of neutrophilic asthma. Neutrophilic asthma is characterized by a rise in neutrophil levels of over 65%, in contrast to eosinophils, which represent less than 2.5% of the total leukocyte count. The results showed a significant increase in leukocytes in both types of samples. Furthermore, there was a notable elevation of neutrophils compared to the control group. However, no statistically significant difference was observed in the percentage of eosinophils in either type of sample. These findings strongly suggest that NTHi OMVs may induce neutrophilic asthma [18]. To delve into the types of receptors responsible for the recognition of OMVs, as well as the areas and organs affected after infection with NTHi, a group of mice was administered with OMVs labeled with the PKH67 fluorophore. Histological analysis indicated that the OMVs were capable of adhering to the respiratory tract epithelium of the mice [18]. 

The above-mentioned studies highlight bacterial-derived OMVs’ role in different disease settings. However, further studies are required to provide mechanistic insights. 

## 6. Antimicrobial Resistance

The function of OMVs has been examined in relation to the effectiveness of antibiotics. When susceptible bacteria are treated with antibiotics, they undergo stress responses, leading to the release of OMVs. Some of these studies include the formation of biofilms as a response to bacterial stress, characterized by the formation of complex biomolecules that contain proteins, lipids, and nucleic acids. This complex matrix has been associated with protecting bacteria against antibiotics and enzymes [63], thereby promoting bacterial survival. 

Studies indicate that OMVs may be essential in neutralizing the effects of antibiotics and other antimicrobial compounds. This is done by acting as decoys that bind and absorb antimicrobial peptides or phages, functioning as physical barriers to protect bacteria from being eliminated [16,64,65].

An example of this characteristic can be observed in OMVs derived from *Stenotrophomonas maltophilia*, which carry enzymes such as β-lactamases. They not only provide protection to *S. maltophilia* against β-lactam antibiotics but also confer resistance to other bacteria, including *Pseudomonas aeruginosa* and *Burkholderia cenocepacia* [66]. Furthermore, it has been observed that antibiotic-resistant bacterial strains, such as *E. coli*, *Moraxella catarrhalis*, and *B. fragilis*, secrete OMVs containing β-lactamase, thus diminishing the effectiveness of β-lactam antibiotics against these strains [67,68,69]. The capacity of OMVs to undermine antibiotic activity extends beyond β-lactams, affecting other antibiotics such as colistin, melittin, and polymyxin B. OMVs, therefore, emerge as critical participants in the degradation of these antimicrobial compounds [70,71,72].

Other studies have also uncovered a novel role of OMVs that is closely associated with antimicrobial resistance (AMR). This mechanism is based on the transfer of antimicrobial resistance genes (ARGs) between bacteria. Antibiotic-susceptible bacteria can acquire ARGs from other bacteria, regardless of whether they belong to the same or a different species, using OMVs as carriers. OMVs are a more recently identified novel delivery mechanism of AMR gene transfer. This mechanism presents an alternative to the more traditional routes of gene transfer, such as natural transformation, transduction, or conjugation by bacterial cells, and overcomes some of their limitations, such as host specificity, a restricted genetic payload, and the type of genetic material transferred [73].

The packaging of genetic material, including ARGs, into OMVs and its transfer occurs through horizontal gene transfer. This facilitates OMV to carry drug resistant genes in microbial communities and, thus, enables bacteria to acquire resistance genes even from distant relatives, leading to the dissemination of resistance among bacterial populations. This mechanism has been reported in several Gram-negative bacteria, including *Acinetobacter baumannii*, *E. coli*, *Porphyromonas gingivalis*, *N. gonorrhoeae*, and *Pseudomonas aeruginosa*. These findings underscore the importance of understanding the roles of OMVs in AMR, as they contribute to the rapid spread of resistance and pose challenges to effectively combating bacterial infections [73,74,75].

Studies have demonstrated the role of OMVs in biofilm formation, through the packaging of molecules with the capability of regulating both the formation and structure biofims. As an example, a study by Zhao and co-workers demonstrated that OMVs isolated and purified from *P. aeruginosa* were packaged with a quorum sensing molecule, *Pseudomonas* quinolone signal (PQS), which regulated biofilm structure and formation, while also changing the microbial diversity within the biofilm community [76]. Biofilm formation has significant implications in terms of AMR, facilitating bacteria evasion from antibiotics used to treat infections. In a further study, Seike and colleagues showed that OMVs released from *Aeromonas* strains were involved in biofilm formation [77]. Specifically, the authors demonstrated that OMVs purified from several *Aeromonas* strains induced biofilm formation in a dose-dependent manner. 

## 7. Production of OMV from Commensal Microbiota

Although OMVs have primarily been associated with the pathogenesis and delivery of virulence factors, recent evidence also suggests a non-pathogenic role of OMVs. Within the gut, commensal bacteria produce OMVs which act as carriers of essential molecules for gut bacteria, contributing to microbiota homeostasis [13]. These mutualistic functions have mainly been explored in members of the genus *Bacteroides*, which release OMVs that influence the physiology of epithelial cells and the host immune response by secreting glycosylases and proteases that aid the degradation of polysaccharides and mucins that can be used as substrates by other bacteria [3,78,79]. 

The intestinal microenvironment represents a crucial and complex ecological system within the host, and is sustained through the interaction between the microbial community and the host’s immune system [80]. For example, it has been demonstrated that *Bacteroides fragilis*-derived OMVs preferentially activate innate immune receptors compared to those of their parent bacteria. Notably, interactions between OMVs of gut bacteria and epithelial cells involve specific components such as adhesins, sulfatases, and proteases [81]. For example, *Bacteroides thetaiotaomicron* (*B*. *thetaiotaomicron*) plays an essential role in harvesting the sugar-rich mucin from the intestinal epithelium, providing accessibility to other bacterial species which are incapable of producing it within the microbiota. Moreover, the sialic acid released by *B. thetaiotaomicron* serves as a growth advantage for both *Clostridioides difficile* and *Salmonella typhimurium* [81]. All of the abovementioned studies highlight the importance of different biomolecules and enzymes carried *via* OMVs in the balance of the gut microbiota and in maintaining health and homeostasis in the host.

Other studies have demonstrated the anti-inflammatory and barrier-protection properties of OMVs derived from commensal bacteria. For instance, *Bacteroides fragilis* releases OMVs that exhibit immunomodulatory and protective effects against colitis. These OMVs, containing polysaccharide A, are identified by dendritic cells through TLR2 activation, promoting T regulatory cell activity and the production of anti-inflammatory cytokines (IL-10) and protecting the host from experimental colitis [3].

A study conducted by Wang et al. (2023) explored the role of OMVs in regulating intestinal balance utilizing other bacterial models. The authors specifically focused on the significance of gut microbiota-derived OMVs, particularly those released by *Akkermansia muciniphila*, in maintaining intestinal health. This bacterium, belonging to the *verrucomicrobia* phylum, is characterized as a common mucin-degrading bacteria found in the human intestinal microbiota [82]. The OMVs released by *Akkermansia muciniphila* have the ability to trigger an immunoglobulin A (IgA) response in the intestinal mucosa by migrating to Peyer’s patches and subsequently activating B cells and dendritic cells. IgA plays a crucial role in preserving the immune barrier in the intestine, protecting it against pathogen invasion. Therefore, it was observed that these OMVs can correct the altered balance of the intestinal microbiota by selectively promoting the growth of beneficial bacteria. This fundamental function helps correct dysbiosis in the intestine, addressing the imbalance in microbiota composition [82].

A comparative study of commensal and pathogenic *E. coli* strains analyzed the immunological response triggered by the release of OMVs. The study demonstrated that the nature and intensity of these responses differ significantly between the two types, suggesting a crucial role in determining the balance between inflammation and intestinal homeostasis. Upon the production and release of OMVs by pathogenic *E. coli*, there was a significant increase in the expression of pro-inflammatory cytokines compared to that of the non-pathogenic strain. This indicated a potential for the pathogenic strain’s OMVs to induce inflammation, disrupt tight junctions of epithelial cells, and damage underlying cells. On the other hand, OMVs released by the non-pathogenic *E. coli* strain appeared to influence intestinal homeostasis by inducing anti-inflammatory responses [13].

In light of these discoveries, it is evident that packaging bioactive macromolecules into OMVs in gut bacteria enables a mutually beneficial relationship between the bacteria and the host. This characteristic has been found in a diverse range of bacterial species. This knowledge paves the way for studying OMVs as a novel mechanism for transporting molecules of interest, such as proteins, throughout the body [82,83]. However, one of the major challenges in the study of the beneficial role of OMVs between commensal bacteria and the host is the change in and composition of OMVs following changes in or dysbiosis of the intestinal microbiota. Comprehending these mechanisms is essential to understanding the role of OMVs in the long-distance transport of biomolecules to the host and requires further detailed investigations [84].

## 8. Outlook and Future Direction

OMVs have emerged as a promising area of research in recent years, garnering significant attention due to their diverse biological functions and potential applications. The multifaceted functions of OMVs play a crucial role in the survival of Gram-negative bacteria, exerting a significant influence on bacterial ecology. Understanding the biogenesis, ecological role, genetic basis, and precise stimulation pathway of OMVs is essential for achieving enhanced yields and developing optimal products to combat bacterial pathogens effectively [82].

One promising area that necessitates close examination is unraveling the molecular mechanisms involved in OMVs’ biogenesis and cargo selection. While significant advances have been made in comprehending the biogenesis process, numerous questions persist. OMVs are thought to bud from the outer membrane, encapsulating diverse biomolecules, including lipids, proteins, and nucleic acids such as phosphatidylethanolamine, phosphatidylglycerol, porin proteins, and sRNAs like *MicF* from *E. coli.* However, further exploration is pivotal to comprehensively elucidate the intricate machinery and regulatory factors orchestrating this process. As mentioned by Schwechheimer and Kuehn (2015), this knowledge is crucial in devising strategies to modulate OMVs’ production for therapeutic or diagnostic purposes [20].

Furthermore, comprehending OMVs’ role in bacterial pathogenesis remains an area that needs to be further elucidated. While recent studies have shed light on OMVs serving as potent carriers for virulence factors, toxins, and immune evasion molecules, the precise molecular mechanisms through which OMVs facilitate bacterial pathogenicity, including their interactions with host cells, continue to be the subject of ongoing investigation. Additionally, research must persist in unraveling the host responses triggered by OMVs, as these responses are likely to hold key insights into disease progression [10,14]. 

In the context of disease settings, OMVs are progressively gaining recognition for their diagnostic and therapeutic potential. OMVs sourced from pathogenic bacteria may harbor specific antigens that can be employed for vaccine development. A number of recently published reviews have comprehensively reviewed the potential of bacterial OMVs as bacterial-derived vaccination systems [85,86]. Furthermore, OMVs exhibit promise as delivery vehicles for various drug compounds for illness treatments [63].

One of the most significant biotechnological applications of OMVs is their use as vaccines, which has been extensively studied in animals and humans for several decades, particularly in combating diseases caused by *Neisseria meningitis* and *N. gonorrhoeae* [19,87]. The need for an effective vaccine formulation has prompted the search for ideal adjuvants to enhance vaccine efficacy. Over time, numerous successful experiments have developed OMVs with demonstrated safety and immune-stimulating properties, making them promising candidates for therapeutic applications as adjuvant tools. OMVs show potential as carriers and adjuvants for nasal vaccines, providing a novel approach to improve immunization strategies [88,89].

Researchers are currently directing their efforts toward the development of OMV-based vaccines, with a focus on manipulating their protein and endotoxin levels to modulate their immunogenicity and toxicity. Significant progress has been made towards the utilization of OMVs as vaccines candidates, specifically with respect to NTHi. The findings of Roier et al. (2012) revealed a robust and complex immune response following nasopharyngeal immunization of mice with OMVs derived from different NTHi strains. The researchers identified vital immunogenic proteins and various OMPs through immunoprecipitation. Surprisingly, the induced immune response exhibited cross-protective efficacy, providing protection not only against colonization with the homologous NTHi strain used for immunization but also against heterologous NTHi strains. These findings hold promising implications for developing an effective vaccine against NTHi infections and other pathogens, thereby contributing to advancing preventive strategies and public health interventions [16]. This offers new possibilities for enhancing vaccine development. By targeting specific host factors triggered by OMVs, it may be possible to reduce tissue-damaging inflammation during infections. Furthermore, inflammation is intricately linked to metabolic pathways, offering novel opportunities to intervene and influence immune responses effectively [90]. 

## 9. Conclusions

The emerging understanding of OMVs has unveiled their pivotal role in the pathogenicity of infectious diseases, shedding light on their interactions with host cells, their contributions to inflammation, and their involvement in AMR. Conversely, while numerous OMVs are associated with pathogenic bacteria and the exacerbation of inflammatory disease, OMVs also help maintain health and homeostasis. For example, OMVs produced within the gut play a crucial role in maintaining a balanced gut microbiota, fostering symbiotic relationships that significantly contribute to the overall health and well-being of the host. This review has discussed OMVs’ structure and biogenesis mechanisms, as well as the role of OMVs in pathogenesis and AMR, while also alluding to their beneficial properties in maintaining homeostasis. While recent research efforts have granted insight into both the pathogenic and beneficial functions of OMVs, further research into the therapeutic applications of OMVs, for example, as vaccine candidates, is warranted.
